# Hospitalizations due to unintentional transport injuries among Aboriginal population of British Columbia, Canada: Incidence, changes over time and ecological analysis of risk markers

**DOI:** 10.1371/journal.pone.0191384

**Published:** 2018-01-26

**Authors:** Mariana Brussoni, M. Anne George, Andrew Jin, Ofer Amram, Rod McCormick, Christopher E. Lalonde

**Affiliations:** 1 Department of Pediatrics, University of British Columbia, Vancouver, British Columbia, Canada; 2 School of Population and Public Health, University of British Columbia, Vancouver, British Columbia, Canada; 3 British Columbia Children’s Hospital Research Institute, Vancouver, British Columbia, Canada; 4 British Columbia Injury Research & Prevention Unit, Vancouver, British Columbia, Canada; 5 Epidemiology Consultant, Surrey, British Columbia, Canada; 6 Department of Nutrition and Exercise Physiology, Elson S. Floyd College of Medicine, Washington State University, Vancouver, British Columbia, Canada; 7 Faculty of Human, Social and Educational Development, Thompson Rivers University, Kamloops, British Columbia, Canada; 8 Department of Psychology, University of Victoria, Victoria, British Columbia, Canada; University of California San Francisco, UNITED STATES

## Abstract

**Background:**

Worldwide, Indigenous people have disproportionately higher rates of transport injuries. We examined disparities in injury-related hospitalizations resulting from transport incidents for three population groups in British Columbia (BC): total population, Aboriginal off-reserve, and Aboriginal on-reserve populations. We also examined sociodemographic, geographic and ethnic risk markers for disparities.

**Methods:**

We identified Aboriginal people through BC’s universal health care insurance plan insurance premium group and birth and death record notations. We calculated crude incidence rate and Standardized Relative Risk (SRR) of hospitalization for unintentional transport injury, standardized for age, gender and Health Service Delivery Area (HSDA), relative to the total population of BC. We tested hypothesized associations of geographic, socio-economic, and employment-related characteristics of Aboriginal communities with SRR of transport injury by multivariable linear regression.

**Results:**

During the period 1991–2010, the SRR for the off-reserve Aboriginal population was 1.77 (95% CI: 1.71 to 1.83); and 2.00 (95% CI: 1.93 to 2.07) among those living on-reserve. Decline in crude rate and SRRs was observed over this period among both the Aboriginal and total populations of BC, but was proportionally greater among the Aboriginal population. The best-fitting multivariable risk marker model was an excellent fit (R^2^ = 0.912, p<0.001), predicted SRRs very close to observed values, and retained the following terms: urban residence, population per room, proportion of the population with a high school certificate, proportion of the population employed; and multiplicative interactions of Aboriginal ethnicity with population per room and proportion of the population employed.

**Conclusions:**

Disparities in risk of hospitalization due to unintentional transport injury have narrowed. Aboriginal ethnicity modifies the effects of socioeconomic risk factors. Continued improvement of socioeconomic conditions and implementation of culturally relevant injury prevention interventions are needed.

## Introduction

Injuries on the world’s roads kill more than 1.25 million people annually and are the leading cause of death for 15-29-year-olds [1]. This epidemic prompted the World Health Organization to launch the Decade of Action for Road Safety 2011–2020, and in April 2016, the United Nations General Assembly adopted Sustainable Development Goals targeting reducing road injuries by 50% by 2020.

In Canada, transport injuries represent a major economic burden, costing $4.289 billion in direct and indirect costs in 2010 [2]. These costs resulted, in part, from the death of 2,620 Canadians and hospitalization of 28,350. Transport incidents include those involving a variety of means of transportation, such as cars, motorcycles, trucks, buses, boats, airplanes, all-terrain vehicles, bicycles and walking; with collisions involving bicycles, pedestrians, and cars being the most common cause of transport-related injuries, accounting for 50% of total incidents [2].

British Columbia’s (BC) age and sex standardized transport death rate is higher than the Canadian average (7.7 versus 7.2 per 100,000, respectively) [2]. In 2010, transport-related injuries represented an economic burden of $658 million for BC, and were the most expensive to treat of all leading causes of injury, with each hospitalization costing an average of $22,393 [3].

Canadian and international research has identified that some populations are at greater risk of transport injuries than others, particularly young adult males, rural populations, and Aboriginal populations [1,4–16]. Canada’s Aboriginal populations, includes First Nations, Inuit and Métis peoples. In previous research, we highlighted disparities in injury rates between Aboriginal and non-Aboriginal populations in BC, including those related to unintentional transport incidents [17–26]. Understanding causal pathways for these disparities is necessary in order to identify potential risk factors that enable public health and policy efforts to tackle them.

In this report, we examine injury rate differences for hospitalizations related to unintentional transport injuries among the total population of BC compared to Aboriginal populations living on- and off-reserve, differences in rates by gender, age and metropolitan or non-metropolitan geography, as well as time trends. Furthermore, we test the hypothesis that disparities of risk between Aboriginal and total populations and between Aboriginal populations living on- and off-reserve are attributable to socioeconomic status, geographic place (urban-rural continuum), and Aboriginal ethnicity.

## Methods

### Ethics review and permission for data access

The University of British Columbia Behavioural Research Ethics Board reviewed our methods (BREB file H06-80585). Data Stewards representing the BC Ministry of Health and the BC Vital Statistics Agency approved the data access requests. We used existing databases, permanently linked by British Columbia Personal Health Number, maintained by Population Data BC [27–30]. These data are available on request, from Population Data BC (https://www.popdata.bc.ca/data, contact: Kelly Sanderson, Researcher Liaison Unit Lead, e-mail: kelly.sanderson@popdata.bc.ca; specifications in project file George 11–012) subject to approval by the Data Stewards representing the British Columbia Ministry of Health Services, and the Vital Statistics Agency of British Columbia, for ethical and privacy reasons, because the data pertain to individuals. The data may be accessed and statistically analysed only on Population Data BC’s Secure Research Environment cloud server. **Disclaimer**: All inferences, opinions, and conclusions drawn in this report are those of the authors, and do not reflect the opinions or policies of the Data Stewards.

### Population counts

This study used methods described in previous publications [17–19, 21–26]. We used the registration and premium billing files [27] of the province’s universal health care insurance program as a population registry. We identified “Aboriginal” persons by insurance premium group and notations on linked Vital Statistics birth records [29] and death records [28]. We used postal code of residence to classify Aboriginal people as residing "on-reserve" or "off-reserve”, and to assign people to Health Service Delivery Areas (HSDAs) [31]. We classified HSDAs within Vancouver and Victoria (the two largest Census Metropolitan Areas in BC) as “metropolitan” and all other HSDAs as “not metropolitan”

### Hospitalization counts

We tabulated hospital separations [30] among residents of BC, occurring from April 1, 1991 through March 31, 2010. We considered a hospitalization as “due to injury” if the level of care was “acute” or “rehabilitation”, and the Most Responsible Diagnosis on the discharge record was an International Classification of Diseases Revision 9 (“ICD-9”) numeric code in the range 800 through 999, or an International Classification of Diseases Revision 10 (“ICD-10”) code in the range S00 through T98. We considered an injury as “unintentional (and due to) transportation” if the first occurrence of the supplemental injury diagnosis code (indicating intention and external cause) was an ICD-9 E-code in the ranges E800-E807, E810-E829, E831, E833-E838, or E840-E848, or an ICD-10 code in the ranges V01-V89, V91, or V93-V99. Captured within these codes are injuries to pedestrians, cyclists, occupants in all motorized vehicles (motorcycles, cars, trucks, buses), off-road (e.g., all-terrain vehicles), boats and airplanes.

### Incidence rates of hospitalization

As in previously reported analyses [17–19, 21–26], we calculated **crude rate** and **Standardized Relative Risk** (SRR) of hospitalization (relative to the total population of BC during the same time period) using the indirect method of standardization [32]. We standardized by gender, 5-year age group and HSDA when comparing population groups, but standardized by gender and 5-year age group when comparing HSDAs. The SRR could also be called the Standardized Incidence Ratio.

### Predictors of risk

We studied risk markers for hospitalization due to injury, using an ecological approach, where the unit of observation was the HSDA (n = 16) subdivided into three population groups (total population, Aboriginal off-reserve, and Aboriginal on-reserve) and two time periods (1999–2003, and 2004–2008). As hypothesized risk markers, we selected socio-economic, housing, and geographic indicators previously developed by Statistics Canada and Aboriginal Affairs and Northern Development Canada. We previously published definitions and reasons for selection of the markers [18,21,23–26]. The descriptive profiles of these variables relating to the three population groups have been previously published [18,24,26].

The effects of socioeconomic and geographic risk markers might not be the same for Aboriginal people as for the general population. Therefore, we created ethnicity interaction variables, calculated as each of the socioeconomic or geographic risk markers multiplied by the proportion of the population who were Aboriginal (0≤*Aboriginal*≤1).

### Ecological analysis

For each HSDA sub-population, we calculated the age and gender standardized SRR of hospitalization due to unintentional transportation injury during the period 1999 through 2003 (a 5-year period centred about the Census year 2001) and during the period 2004 through 2008 (centred about the Census year 2006), relative to the total population of BC during the same time period. The natural logarithm of SRR (LnSRR) appeared to be normally distributed (Shapiro-Wilk statistic 0.982, df = 91, p = 0.261, consistent with the null hypothesis that LnSRR is randomly sampled from a normally distributed population); therefore, we used LnSRR as the dependent (Y) variable for regression analysis. Note that for purposes of the ecological analysis, from 96 (16x2x3) potential data points we excluded five where LnSRR was undefined: four with on-reserve Aboriginal population of zero, and one where zero injury hospitalizations occurred among its very small on-reserve Aboriginal population. We performed statistical analyses with IBM SPSS Statistics, Version 19.

As in previously published analyses [18,21,23–26], we tested hypotheses of association by performing linear regressions, weighted by person-years to diminish the impact of extreme values of LnSRR occurring in smaller population units. We modelled the relationship of LnSRR and the independent (X) variables as LnSRR = B•*x* + Constant. The regression coefficient "B" represents the hypothesized effect on LnSRR of unit change in *x*. We modeled the relationship of LnSRR and the interactions of Aboriginal with each of the independent variables as LnSRR = B•*Aboriginal*•*x* + Constant. The regression coefficient "B" of the interaction term *Aboriginal*•*x* represents the hypothesized effect on LnSRR of unit change in *x* among the Aboriginal portion of the population.

We tested census year, hypothesized socio-economic, work-related, geographic, and ethnicity markers, and interaction terms in turn as the single independent variable, then we used stepwise forwards selection of variables to arrive at the best-fitting multivariable model. In the final model, we tested the normality of the distribution of the standardized residuals by the Shapiro-Wilk statistic, and we verified homoscedasticity by scatter-plotting the standardized residuals against the regression-predicted values of LnSRR.

To verify that the step-wise regression procedure (weighted by population) had produced a model representative of the experience of the total population of BC and the two much smaller Aboriginal populations as well, we used the final regression model as a risk prediction calculator, comparing the predicted to the observed disparities of injury SRR among the three population groups.

## Results

### Aboriginal status and reserve residence

The crude rates and SRRs of hospitalization for unintentional transport injuries from 1991–2010 for BC’s total population (i.e., the reference population), Aboriginal population, and the Aboriginal population residing on- or off- reserve can be seen in Table 1. The Aboriginal population has higher crude rates than the total population, with those living on reserve experiencing the highest risk, with a SRR double that of BC’s total population.

**Table 1:** Hospital Separations due to unintentional transport injuries*, British Columbia, 1991–2010†.

	Person-years^‡^	Observed injuries^§^	Expected injuries^¶^	Crude Rate^\\^	95% CI for Crude Rate			SRR**	95% CI for SRR		
BC, total population	78,256,306	262,819	262,818	33.6	33.5	-	33.7	1	[reference]		
BC, Aboriginal	2,541,060	12,683	6,710	49.9	49.1	-	50.8	1.89	1.85	-	1.94
BC, Aboriginal, off-reserve	1,403,813	5,810	3,277	41.4	40.3	-	42.5	1.77	1.71	-	1.83
BC, Aboriginal, on-reserve	1,131,862	6,839	3,421	60.4	59.0	-	61.9	2.00	1.93	-	2.07

* "Unintentional transportation injury" defined as hospital separation with Most Responsible Diagnosis in the range ICD9:800–999 or ICD10:S00-T98, and supplemental diagnosis in the range ICD9:E800-E807, E810-E829, E831, E833-E838, E840-E848 or ICD10:V01-V89, V91, V93-V99.

^**†**^ Injuries occurring during the observation period 1991-Apr-01 to 2010-Mar-31.

^**‡**^ Person-years is the sum of the annual population counts times the fraction of each year included in the observation period.

^**§**^ Observed number of injuries.

^**¶**^ Expected number of injuries, indirectly standardized, based on age, gender and HSDA-specific rates in the total population of BC.

^**\\**^ Crude Rate per 10,000 person-years.

** Standardized Relative Risk (compared to the total population of BC) = Observed/Expected.

### HSDAs and urban residence

**Fig 1:**
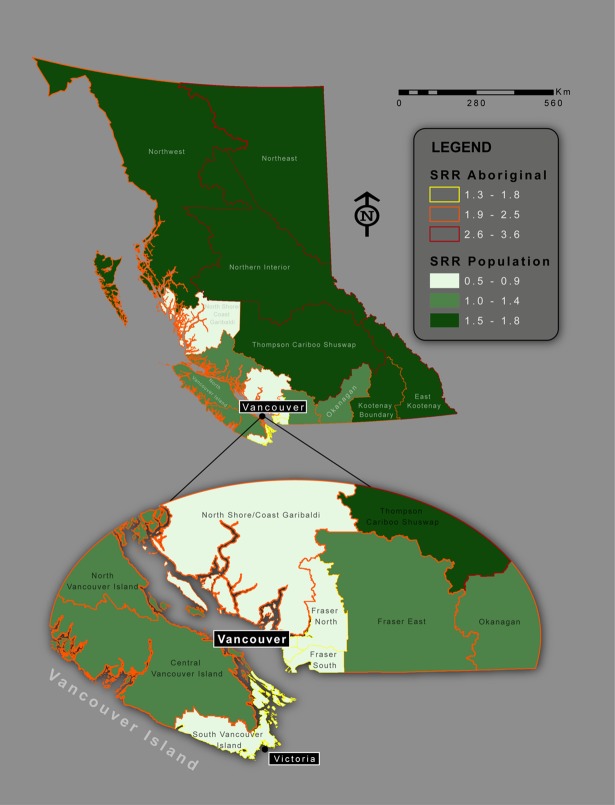
SRR of hospital separations due to unintentional transport injuries by HSDA, British Columbia, 1991–2010.

Table 2 and Fig 1 show HSDA differences in crude rates and age and gender-standardized SRRs for hospitalizations resulting from unintentional transport injuries in BC from 1991–2010, for BC’s total Population and Aboriginal population. The anomalously low SRR in the large but thinly populated mainland coastal area north of Vancouver Island is an artifact that appears because this area is counted as part of metropolitan HSDA 33. These data indicate that populations living in metropolitan HSDAs, regardless of ethnicity, experienced lower crude rates and SRRs. Populations living in non-metropolitan HSDAs had a SRR 1.96 times greater than those living in metropolitan HSDAs (1.43 vs. 0.73). Overall, Aboriginal populations experienced higher SRRs than the total population, regardless of the HSDA they resided in and whether it was metropolitan or not. The disparity in injury rates between Aboriginal populations residing in metropolitan and non-metropolitan areas was evident (SRR 1.57 times higher for the latter group, i.e., 2.71 vs 1.73), but not as pronounced as in the total population.

**Table 2:** Hospital separations for unintentional transport injuries*, British Columbia, 1991–2010†, by Health Service Delivery Area.

	Total population									Aboriginal population								
HSDA	Observed injuries^‡^	Crude Rate^§^	95% CI for Crude Rate			SRR^¶^	95% CI for SRR			Observed injuries^‡^	Crude Rate^§^	95% CI for Crude Rate			SRR ^¶^	95% CI for SRR		
11	3,504	23	23	-	24	1.70	1.63	-	1.78	126	40	33	-	47	2.98	2.20	-	4.04
12	3,416	22	22	-	23	1.64	1.57	-	1.71	33	29	20	-	40	2.25	1.34	-	3.84
13	9,820	17	16	-	17	1.22	1.20	-	1.25	434	32	29	-	35	2.41	2.08	-	2.79
14	10,271	25	25	-	26	1.83	1.79	-	1.88	1,626	49	47	-	52	3.54	3.23	-	3.88
21	7,004	15	15	-	16	1.12	1.10	-	1.15	442	27	25	-	30	2.02	1.77	-	2.30
22	9,677	10	10	-	10	0.70	0.69	-	0.72	156	16	14	-	19	1.27	1.07	-	1.52
23	12,720	11	11	-	12	0.83	0.82	-	0.85	192	18	16	-	21	1.50	1.26	-	1.78
31	2,195	7	6	-	7	0.48	0.47	-	0.49	24	17	11	-	26	1.33	0.84	-	2.15
32	9,972	9	9	-	9	0.63	0.62	-	0.64	544	25	23	-	27	1.83	1.64	-	2.05
33	6,029	12	12	-	12	0.88	0.86	-	0.91	541	29	26	-	31	2.05	1.82	-	2.31
41	7,131	11	11	-	11	0.82	0.80	-	0.84	278	21	19	-	24	1.58	1.36	-	1.83
42	6,962	15	15	-	16	1.13	1.10	-	1.16	835	31	29	-	33	2.26	2.04	-	2.50
43	4,115	19	18	-	19	1.39	1.34	-	1.44	439	34	31	-	37	2.51	2.16	-	2.91
51	3,889	24	23	-	25	1.73	1.66	-	1.80	1,284	32	31	-	34	2.32	2.14	-	2.53
52	6,783	24	23	-	24	1.69	1.64	-	1.74	991	44	41	-	47	3.18	2.85	-	3.55
53	3,214	26	25	-	27	1.83	1.74	-	1.92	390	47	43	-	52	3.58	2.96	-	4.32
Metro^**\\**^	47,724	10	10	-	10	0.73	0.73	-	0.74	1,735	23	22	-	24	1.73	1.63	-	1.84
Not**	58,978	20	19	-	20	1.43	1.42	-	1.45	6,600	37	36	-	38	2.71	2.61	-	2.82
All HSDAs	106,702	14	14	-	14	1	[reference]			8,335	33	32	-	34	2.42	2.35	-	2.51

* "Unintentional transportation injury" defined as hospital separation with Most Responsible Diagnosis in the range ICD9:800–999 or ICD10:S00-T98, and supplemental diagnosis in the range ICD9:E800-E807, E810-E829, E831, E833-E838, E840-E848or ICD10:V01-V89, V91, V93-V99.

^**†**^ Injuries occurring during the observation period 1991-Apr-01 to 2010-Mar-31.

^**‡**^ Observed number of injuries.

^**§**^ Crude Rate per 10,000 person-years.

^**¶**^ Standardized Relative Risk (indirectly standardized by age and gender, compared to the total population of BC) = Observed/Expected.

^**\\**^ Metropolitan: aggregation of HSDAs 22, 23, 31, 32, 33 and 41 (Vancouver and Victoria Census Metropolitan Areas).

** Not metropolitan: aggregation of HSDAs 11, 12, 13, 14, 21, 42, 43, 51, 52, 53.

### Age and gender

Table 3 shows the crude rates and age, gender and HSDA-standardized SRRs for hospitalizations resulting from unintentional transport injuries in BC from 1991–2010, for females and males within specific age categories, for BC’s total Population and Aboriginal population. Regardless of age or ethnicity, males had higher rates than females, with the highest rates occurring among 20-29-year-old males in the total and Aboriginal population. Among females, the highest rates were evident for 10-19-year-olds in the total population, and 20-29-year-olds in the Aboriginal population.

**Table 3:** Hospital separations for unintentional transport injuries*, British Columbia, 1991–2010†, by gender and age.

		Total population						Aboriginal population								
Gender	Age	Observed injuries^‡^	Crude Rate^§^	95% CI for Crude Rate			SRR [ref]	Observed injuries^‡^	Crude Rate^§^	95% CI for Crude Rate			SRR^¶^	95% CI for SRR		
Female	0–9	2,064	5	4	-	5	1	221	8	7	-	9	1.41	1.21	-	1.65
	10–19	6,789	14	13	-	14	1	606	27	25	-	29	1.55	1.41	-	1.72
	20–29	6,359	12	12	-	12	1	760	38	35	-	40	2.36	2.12	-	2.63
	30–39	5,457	9	9	-	9	1	712	34	32	-	37	2.76	2.44	-	3.12
	40–49	4,908	8	8	-	8	1	453	27	24	-	29	2.51	2.17	-	2.90
	50–59	3,932	8	8	-	9	1	276	27	24	-	31	2.53	2.10	-	3.05
	60–69	2,953	9	9	-	9	1	156	28	24	-	33	2.39	1.87	-	3.04
	70–79	3,062	12	12	-	13	1	74	27	21	-	34	1.82	1.34	-	2.48
	80+	2,098	12	12	-	13	1	25	17	12	-	26	1.32	0.84	-	2.10
	Total	37,622	10	9	-	10	1	3,283	26	25	-	26	2.13	2.03	-	2.24
Male	0–9	3,454	7	7	-	7	1	369	13	11	-	14	1.49	1.32	-	1.69
	10–19	13,928	27	26	-	27	1	1,057	45	43	-	48	1.32	1.23	-	1.41
	20–29	15,784	30	29	-	30	1	1,385	70	67	-	74	1.71	1.60	-	1.83
	30–39	12,083	20	20	-	20	1	1,002	51	47	-	54	1.90	1.74	-	2.07
	40–49	10,096	16	16	-	17	1	687	44	41	-	48	2.07	1.86	-	2.31
	50–59	6,597	14	14	-	14	1	354	39	35	-	43	2.16	1.85	-	2.51
	60–69	3,849	12	11	-	12	1	152	31	27	-	37	2.03	1.62	-	2.55
	70–79	2,823	13	13	-	14	1	80	36	29	-	44	2.16	1.56	-	3.00
	80+	1,659	16	15	-	17	1	18	18	11	-	29	0.94	0.60	-	1.50
	Total	70,273	18	18	-	18	1	5,104	41	40	-	42	1.69	1.63	-	1.75

* "Unintentional transportation injury" defined as hospital separation with Most Responsible Diagnosis in the range ICD9:800–999 or ICD10:S00-T98, and supplemental diagnosis in the range ICD9:E800-E807, E810-E829, E831, E833-E838, E840-E848or ICD10:V01-V89, V91, V93-V99.

^**†**^ Injuries occurring during the observation period 1991-Apr-01 to 2010-Mar-31.

^**‡**^ Observed number of injuries.

^**§**^ Crude Rate per 10,000 person-years.

^**¶**^ Standardized Relative Risk (indirectly standardized by age, gender and HSDA, compared to the total population of BC) = Observed/Expected.

Aboriginal populations showed higher rates of hospitalization resulting from unintentional transport injuries regardless of age or gender, compared to the total population of BC. Larger SRRs were evident for Aboriginal females than for males, indicating greater disparities with the total population. The largest disparity was among Aboriginal females aged 30–39 years, with a SRR 2.76 times that of their counterparts in the total population. Among males, the highest SRRs were evident among 50–59 and 70-79-year-olds.

### Changes over time

Annual crude rates and age, gender and HSDA standardized SRRs of hospitalizations resulting from unintentional transport injuries, during the period 1991–2010, among BC’s total and Aboriginal populations can be seen in Table 4. The reference population is the total population of BC over all the years (1991–2010). Crude rates of unintentional transport injury decreased across time, regardless of ethnicity. While the Aboriginal population experienced higher crude rates and SRRs than the total population every year, disparities decreased over time. In 2010, the SRRs for the total population and Aboriginal populations were 0.52 and 0.57, respectively, with overlapping 95% CIs indicating differences were no longer significant (p = 0.76, 2-sided).

**Table 4:** Hospital separations for unintentional transport injuries*, British Columbia, 1991–2010†, by calendar year.

	Total population									Aboriginal population								
Year	Observed injuries^‡^	Crude Rate^§^	95% CI for Crude Rate			SRR^¶^	95% CI for SRR			Observed injuries^‡^	Crude Rate^§^	95% CI for Crude Rate			SRR^¶^	95% CI for SRR		
1991	5,943	23	23	-	24	1.67	1.61	-	1.72	545	67	62	-	73	3.60	3.07	-	4.22
1992	7,225	21	20	-	21	1.48	1.44	-	1.52	609	54	50	-	59	2.94	2.57	-	3.37
1993	6,891	19	18	-	19	1.36	1.32	-	1.40	580	50	46	-	54	2.72	2.38	-	3.11
1994	6,972	18	18	-	19	1.33	1.29	-	1.37	636	53	49	-	57	2.92	2.56	-	3.33
1995	6,587	17	17	-	17	1.23	1.20	-	1.26	636	52	48	-	56	2.88	2.52	-	3.28
1996	6,070	15	15	-	16	1.10	1.07	-	1.13	523	42	38	-	46	2.32	2.03	-	2.64
1997	5,907	15	14	-	15	1.05	1.02	-	1.08	509	40	37	-	44	2.22	1.95	-	2.53
1998	5,782	14	14	-	15	1.02	0.99	-	1.05	499	39	36	-	42	2.16	1.90	-	2.46
1999	5,700	14	13	-	14	1.00	0.97	-	1.03	441	34	31	-	38	1.90	1.67	-	2.16
2000	5,727	14	14	-	14	1.01	0.98	-	1.03	456	35	32	-	38	1.94	1.71	-	2.21
2001	5,231	13	12	-	13	0.91	0.89	-	0.94	385	29	26	-	32	1.62	1.42	-	1.84
2002	5,011	12	12	-	12	0.86	0.84	-	0.89	363	27	24	-	30	1.50	1.32	-	1.70
2003	5,157	12	12	-	12	0.87	0.85	-	0.90	346	25	22	-	27	1.38	1.22	-	1.56
2004	5,045	12	11	-	12	0.85	0.82	-	0.87	304	21	19	-	24	1.19	1.05	-	1.35
2005	4,909	11	11	-	12	0.82	0.80	-	0.84	294	20	18	-	23	1.13	1.00	-	1.28
2006	5,018	11	11	-	12	0.83	0.81	-	0.85	317	21	19	-	24	1.20	1.06	-	1.35
2007	4,897	11	11	-	11	0.80	0.78	-	0.82	334	22	20	-	25	1.24	1.10	-	1.40
2008	4,610	10	10	-	10	0.75	0.73	-	0.76	318	21	18	-	23	1.17	1.04	-	1.31
2009	4,460	10	9	-	10	0.71	0.70	-	0.73	256	16	14	-	18	0.92	0.82	-	1.04
2010	813	7	7	-	8	0.52	0.50	-	0.55	40	10	7	-	14	0.57	0.45	-	0.72
1991–2010	107,955	14	14	-	14	1	[reference]			8,391	33	32	-	34	1.84	1.79	-	1.89

* "Unintentional transportation injury" defined as hospital separation with Most Responsible Diagnosis in the range ICD9:800–999 or ICD10:S00-T98, and supplemental diagnosis in the range ICD9:E800-E807, E810-E829, E831, E833-E838, E840-E848or ICD10:V01-V89, V91, V93-V99.

^**†**^ Injuries occurring during the observation period 1991-Apr-01 to 2010-Mar-31.

^**‡**^ Observed number of injuries.

^**§**^ Crude Rate per 10,000 person-years.

^**¶**^ Standardized Relative Risk (indirectly standardized by age, gender and HSDA, compared to the total population of BC) = Observed/Expected.

Table 5 shows proportional changes in SRR between 1991 and 2010, for the total and Aboriginal populations, as well as males, females and youth (aged under 25 years). Decreases in SRRs were evident for both sexes and among youth, regardless of ethnicity, but with more substantial decreases occurring among the Aboriginal population. As outlined in our previous article reporting time trends for various injury categories [17], total and Aboriginal populations respectively experienced 68.7% (annualized change of -5.9%, 95% CI: -6.2% to -5.6%) and 84.1% (annualized change of -9.2%, 95% CI: -10.6% to -7.9%) decreases in SRRs between 1991 and 2010. Aboriginal females experienced an 86.1% decline in SRR, compared to the 67.7% decline seen in females in the total population of BC (p = 0.001, 2-sided). Similarly, Aboriginal males experienced an 83.2% decline in SRR (annualized change of 8.9%, 95% CI: 10.6% to -7.3%) compared to 69.3% in males among the total population (annualized change of -6.0%, 95% CI: -6.4% to -5.7%). Youth in both the total and Aboriginal populations experienced the greatest declines, with drops of 84.4% (annualized change of -9.3%, 95% CI: -9.8% to -8.8%) and 91.1% (annualized change of -12.0%, 95% CI: -13.8% to -10.1%), respectively. This might indicate that the decreasing disparity might be due to greater improvements among older adults rather than youths.

**Table 5:** Standardized relative risks of hospitalization for unintentional transport injury, British Columbia, 1991–2010.

Demographic category	1991 Observed injuries^‡^	1991 Crude Rate^§^	SRR^¶^ 1991	2010 Observed injuries	2010 Crude Rate	SRR^¶^ 2010	1991 to 2010% change SRR	p*	Annual % change SRR**	95% CI for Annual % change	
Aboriginal	545	67.0	3.60	40	10.0	0.57	-84.1%	<0.001	-9.2%	-10.6%	-7.9%
Aboriginal, Male	358	90.4	3.58	28	14.2	0.60	-83.2%	0.001	-8.9%	-10.6%	-7.3%
Aboriginal, Female	187	45.3	3.63	12	6.0	0.51	-86.1%	0.001	-9.9%	-12.1%	-7.5%
Aboriginal, age under 25 years	262	62.4	3.29	10	5.3	0.29	-91.1%	0.008	-12.0%	-13.8%	-10.1%
BC	5943	23.2	1.67	813	7.0	0.52	-68.7%		-5.9%	-6.2%	-5.6%
BC, Male	3787	30.0	1.63	507	8.8	0.50	-69.3%		-6.0%	-6.4%	-5.7%
BC, Female	2147	16.5	1.72	306	5.2	0.56	-67.7%		-5.8%	-6.3%	-5.3%
BC, age under 25 years	2743	31.1	2.06	168	5.1	0.32	-84.4%		-9.3%	-9.8%	-8.8%

* probability (2-sided, z-test) that Ln((SRR 2010)/(SRR 1991)) Aboriginal = Ln((SRR 2010)/(SRR 1991)) BC.

^‡^ Observed number of injuries.

^§^ Crude Rate per 10,000 person-years.

^¶^SRR: Standardized Relative Risk (indirectly standardized by age, gender and HSDA, compared to the total population of BC, 1991 to 2010) = Observed/Expected.

**Annualized change = (SRR_2010_/SRR_1991_)^1/(2010–1991)^-1.

S1 Table shows that similar patterns of change over time occurred in almost all subsumed categories of unintentional transportation injury, though in most categories, the disparities of decline between the Aboriginal and total populations were not statistically significant, due to small numbers of events in the Aboriginal population. The exception was injury due to off-road motor vehicles, where there was no significant decline of incidence between 1991 and 2010, in either the Aboriginal or the total populations.

### Ecological analysis of predictors of risk

Table 6 shows results from the preliminary regression models including all three population groups with one independent (X) variable, i.e., Ln*SRR* = B*x* + *Constant*. The regression coefficient (*B*) and the “RR Ratio per SD” describe the association between the X variable and transport injury risk in the total population. One SD change in the independent variable is associated with change in the SRR of injury by this factor. For example, one SD change in Income Per Capita is associated with change in SRR by a factor of 0.777 (a reduction, 95% CI = 0.693 to 0.870). P, the probability that R^2^ is equal to zero, was less than 0.05 for all independent variables, except Census (as a proxy for the effect of time) and Labour Force participation.

**Table 6:** Ecologic analysis* of risk of hospitalization due to unintentional transport injury among Health Service Delivery Area Population Groups in British Columbia, 1999–2008†.

X Variable	min	max	mean^‡^	SD^‡^	N	R^2^	B^§^	SE^¶^	p^\\^	RR per SD**	95% CI for RR per SD	
Census	2001	2006	2003.5	2.5	91	<0.001	0.001	0.017	0.975	1.001	0.921	1.089
Income Per Capita ($1,000s)	7.7	36.0	17.1	6.4	91	0.180	-0.040	0.009	<0.001	0.777	0.693	0.870
Income Score	45.1	96.5	69.5	12.4	91	0.207	-0.029	0.006	<0.001	0.700	0.604	0.811
High School	0.315	0.907	0.650	0.132	91	0.298	-2.646	0.430	<0.001	0.704	0.629	0.789
University Degree	0.000	0.364	0.084	0.076	91	0.531	-3.836	0.380	<0.001	0.748	0.706	0.792
Population Per Room	0.403	0.812	0.549	0.097	91	0.151	-2.982	0.749	<0.001	0.748	0.648	0.865
Need Major Repairs	0.050	0.478	0.186	0.116	91	0.218	4.476	0.898	<0.001	1.681	1.367	2.068
Labour Force	0.515	0.771	0.664	0.053	91	0.030	-1.773	1.075	0.103	0.910	0.812	1.020
Employed	0.380	0.734	0.572	0.083	91	0.185	-3.978	0.884	<0.001	0.718	0.620	0.831
Occupation Risk	0.805	1.446	1.111	0.146	91	0.670	2.230	0.166	<0.001	1.386	1.321	1.455
Industry Risk	0.687	1.258	1.064	0.108	91	0.571	2.759	0.253	<0.001	1.348	1.276	1.423
Occupation Risk•Employed	0.350	0.934	0.635	0.126	91	0.350	2.553	0.369	<0.001	1.378	1.257	1.510
Industry Risk•Employed	0.299	0.826	0.609	0.113	91	0.191	2.396	0.522	<0.001	1.310	1.165	1.472
Occupation Risk•Labour Force	0.510	1.055	0.739	0.124	91	0.490	2.665	0.288	<0.001	1.391	1.296	1.493
Industry Risk•Labour Force	0.448	0.900	0.708	0.102	91	0.349	2.876	0.416	<0.001	1.340	1.232	1.458
Urban	0.000	1.000	0.386	0.416	91	0.779	-0.885	0.050	<0.001	0.692	0.664	0.721
Rural	0.000	0.446	0.228	0.153	91	0.837	2.541	0.119	<0.001	1.474	1.422	1.529
Aboriginal	0.007	1.010	0.676	0.447	91	0.259	1.159	0.208	<0.001	1.679	1.396	2.019
NA Indian	0.004	0.992	0.501	0.377	91	0.238	1.409	0.267	<0.001	1.701	1.393	2.077

* The dependent (Y) variable is LnSRR of hospitalization due to unintentional transportation injury, and regression is weighted by person-years.

^†^ Three population groups (total, Aboriginal on-reserve and Aboriginal off-reserve) divided by 16 HSDAs and 2 time periods (1998–2003 and 2004–2008).

^‡^ Unweighted mean and standard deviation (SD) of the independent (X) variable.

^§^ B = regression coefficient.

^¶^ SE = standard error of the regression coefficient.

^\\^ p = probability that B = 0.

** Relative Risk per SD = Exp(BxSD).

Table 7 includes the multiplicative interaction of Aboriginal ethnicity with one each of the variables in Table 6, i.e., Ln*SRR* = B*x*•*Ab* + *Constant*, where *Ab* is the proportion of the population who are Aboriginal. This table describes the associations between each of the independent variables and transport injury risk in the Aboriginal population. Only the variable Urban was non-significantly associated with transport injury risk among the Aboriginal population.

**Table 7:** Ecologic analysis* of risk of hospitalization due to unintentional transport injury among Health Service Delivery Area Population Groups in British Columbia, 1999–2008†.

X•Aboriginal interaction term	min	max	mean^‡^	SD‡	N	R^2^	B^§^	SE^¶^	p^\\^	RR per SD**	95% CI for RR per SD	
Census•Aboriginal	14	2026	1355	896	91	0.259	0.001	<0.001	<0.001	1.679	1.396	2.019
Income Per Capita• Aboriginal	0.2	22.0	9.2	6.2	91	0.321	0.094	0.014	<0.001	1.787	1.496	2.134
Income Score• Aboriginal	0.6	80.1	42.5	28.0	91	0.289	0.020	0.003	<0.001	1.726	1.442	2.068
High School•Aboriginal	0.005	0.871	0.405	0.279	91	0.259	1.924	0.345	<0.001	1.710	1.413	2.070
University Degree• Aboriginal	0.000	0.149	0.032	0.032	91	0.165	17.354	4.119	<0.001	1.746	1.343	2.271
Population Per Room• Aboriginal	0.004	0.812	0.398	0.279	91	0.221	1.764	0.351	<0.001	1.635	1.346	1.985
Need Major Repairs• Aboriginal	0.000	0.478	0.158	0.143	91	0.163	3.317	0.795	<0.001	1.606	1.281	2.012
Labour Force•Aboriginal	0.005	0.758	0.451	0.301	91	0.257	1.734	0.313	<0.001	1.686	1.398	2.033
Employed•Aboriginal	0.004	0.714	0.373	0.254	91	0.261	2.141	0.382	<0.001	1.723	1.421	2.090
Occupation Risk• Aboriginal	0.006	1.446	0.770	0.521	91	0.272	1.015	0.176	<0.001	1.697	1.415	2.037
Industry Risk•Aboriginal	0.007	1.258	0.726	0.487	91	0.272	1.081	0.187	<0.001	1.692	1.412	2.028
Occupation Risk• Employed•Aboriginal	0.004	0.934	0.426	0.298	91	0.275	1.874	0.322	<0.001	1.749	1.445	2.117
Industry Risk• Employed•Aboriginal	0.004	0.826	0.402	0.280	91	0.275	1.991	0.343	<0.001	1.747	1.444	2.114
Occupation Risk• LabourForce• Aboriginal	0.004	1.055	0.514	0.353	91	0.269	1.509	0.264	<0.001	1.704	1.416	2.051
Industry Risk• LabourForce•Aboriginal	0.004	0.859	0.485	0.331	91	0.269	1.606	0.281	<0.001	1.701	1.414	2.045
Urban•Aboriginal	0.000	1.004	0.253	0.378	91	0.007	0.379	0.473	0.425	1.154	0.809	1.647
Rural•Aboriginal	0.000	0.447	0.157	0.158	91	0.319	4.153	0.643	<0.001	1.925	1.573	2.354

* The dependent (Y) variable is LnSRR of hospitalization due to unintentional transportation injury, and regression is weighted by person-years.

^†^ Three population groups (total, Aboriginal on-reserve and Aboriginal off-reserve) divided by 16 HSDAs and 2 time periods (1998–2003 and 2004–2008).

^‡^ Unweighted mean and standard deviation (SD) of the independent (X) variable.

^§^ B = regression coefficient.

^¶^ SE = standard error of the regression coefficient.

^\\^ p = probability that B = 0.

** Relative Risk per SD = Exp(BxSD). One SD change in the independent variable is associated with change in the Standardized Relative Risk of injury by this factor.

The significant variables from Tables 6 and 7 were included in multivariable analyses. Table 8 shows the best-fitting regression model with multiple independent variables remaining after stepwise addition and elimination of the candidate variables (in Table 6) and interaction terms (in Table 7):

$$\mathrm{Ln}\mathrm{SRR}=B_{1}x_{1}+B_{2}x_{2}+B_{3}x_{3}+B_{4}x_{4}+B_{5}x_{5}•\mathrm{Ab}+B_{6}x_{6}•Ab+\mathrm{Constant},$$

where:

*Ab* = proportion of population who are Aboriginal,

x_1_ = proportion of HSDA population residing in large urban centre,

x_2_ = average number of persons per room,

x_3_ = proportion of population, age 25+ years with at least a high school certificate,

x_4_ = proportion of population, age 25+ years, employed,

x_5_ = average number of persons per room,

x_6_ = proportion of population, age 25+ years, employed.

**Table 8:** Ecologic analysis* of risk of hospitalization due to unintentional transport injury among Health Service Delivery Area Population Groups in British Columbia, 1999–2008†.

X Variable	min	max	mean^‡^	SD^‡^	N	B^§^	SE^\\^	p**	RR per SD^††^	95% CI for RR per SD	
(Constant)					91	1.315	0.306	<0.001			
Urban	0.000	1.000	0.386	0.416	91	-0.577	0.054	<0.001	0.787	0.752	0.823
PopPerRoom	0.403	0.812	0.549	0.097	91	-3.662	0.460	<0.001	0.701	0.641	0.766
HighSchool	0.315	0.907	0.650	0.132	91	-1.115	0.226	<0.001	0.863	0.813	0.916
Employed	0.380	0.734	0.572	0.083	91	2.528	0.455	<0.001	1.234	1.145	1.331
PopPerRoom•Aboriginal	0.004	0.812	0.398	0.279	91	3.609	0.600	<0.001	2.735	1.961	3.814
Employed•Aboriginal	0.004	0.714	0.373	0.254	91	-1.988	0.589	0.001	0.603	0.448	0.813

* The dependent (Y) variable is LnSRR of hospitalization due to unintentional transportation injury, and regression is weighted by person-years.

^†^ Three population groups (total, Aboriginal on-reserve and Aboriginal off-reserve) divided by 16 HSDAs and 2 time periods (1998–2003 and 2004–2008).

^‡^ Unweighted mean and standard deviation (SD) of the independent (X) variable.

^§^ B = regression coefficient.

^\\^ SE = standard error of the regression coefficient.

** p = probability that B = 0.

^††^ Relative Risk per SD = Exp(BxSD). One SD change in the independent variable is associated with change in the SRR of injury by this factor.

The best-fitting model was an excellent fit, explaining 91.2% of the variance in unintentional transport injury risk among population groups within HSDAs (R^2^ = 0.912, p<0.001). Higher proportion of urban residence, higher number of persons per room, higher proportion of the population with at least a high school certificate, and lower proportion of the population being employed decreased transport injury risk. Notably, for the Aboriginal population, *lower* number of persons per room and *higher* employment decreased transport injury risk.

Table 9 shows the relative risk predicted by the best-fitting regression model (as seen in Table 8), and the risk-marker characteristics of the Aboriginal off-reserve and on-reserve populations (described in Methods). The direction of the relationships between the independent variables and transport injury risk is the same for off- and on-reserve Aboriginal populations, but of greater magnitude for the latter group.

**Table 9:** Relative risks predicted by the best-fitting multivariable regression model.

	Total Population		Off-Reserve Aboriginal					On-Reserve Aboriginal				
X Variable	Mean*	RR	Mean*	Difference^†^	RR^‡^	95% CI		Mean*	Difference ^†^	RR^‡^	95% CI	
Urban	0.612	1	0.373	-0.239	1.15	1.12	1.18	0.216	-0.396	1.26	1.20	1.31
Population Per Room	0.475	1	0.534	0.059	0.81	0.76	0.85	0.680	0.205	0.47	0.39	0.57
High School	0.778	1	0.658	-0.120	1.14	1.08	1.21	0.513	-0.265	1.34	1.19	1.51
Employed	0.618	1	0.590	-0.027	0.93	0.91	0.96	0.473	-0.145	0.69	0.61	0.79
Population Per Room•Aboriginal	0.022	1	0.534	0.512	6.34	3.44	11.68	0.680	0.658	10.74	4.90	23.54
Employed• Aboriginal	0.028	1	0.590	0.562	0.33	0.17	0.63	0.473	0.444	0.41	0.25	0.70
**Total (product)**		**1**			**2.05**					**2.46**		

* Population-weighted mean of the x-variable, 2001 and 2006 Census, for the specified population group.

^†^ Difference between mean of the specified population group and mean of the total population.

‡ Predicted relative risk associated with the difference, calculated as Exp(B x difference), where B is the regression coefficient in the best-fitting multivariable model.

This model predicts that the off-reserve and on-reserve Aboriginal populations will respectively have a transport injury risk 2.05 times and 2.46 times higher than the total population. These predicted values are higher than the observed SRRs as reported in Table 1 (1.77 and 2.00, respectively). However, the SRRs reported in Table 1 were adjusted for age, gender and HSDA. When adjusted for just age and gender, the SRR for off-reserve Aboriginal populations is 2.06 for the years around the first census period (1999–2003) and 1.90 for the years surrounding the second census period (2004–2008). The SRRs for on-reserve Aboriginal populations are 2.80 and 2.23, respectively. These SRRs are very close to the predicted values reported in Table 9.

## Discussion

Consistent with previous Canadian research examining transport injuries [16], our research confirmed disparities in unintentional transport injury hospitalization rates between the Aboriginal populations and the total populations of BC, regardless of age, gender or geography. The highest rate and largest disparities were evident for Aboriginal peoples living on reserve; and while males aged 20–29 experienced the highest rate of transport injuries, the greatest disparities were evident for females aged 30–39 years. Time trends indicate decreasing rates of transport injury for all of BC, but most pronounced for the Aboriginal population. A number of road safety measures were implemented in BC over the time frame included in our study that have contributed to reducing motor vehicle crashes: requiring daytime running lights on vehicles, graduated licensing program, changes to the Canadian Criminal Code to facilitate detection of impaired driving, and legislation requiring the use of child safety seats [33]. In addition, BC introduced more stringent impaired driving laws in 2010 that have contributed to significant decreases in motor vehicle crashes since our study period ended [34,35]. Over and above these safety initiatives targeting the entire population, there is a need for culturally relevant interventions. Ishikawa et al.’s systematic review of interventions to promote child passenger safety in Aboriginal communities found strong evidence that multicomponent interventions that were community-driven, incorporated their views of health and were tailored to local circumstances and cultures had greater effect [36].

We examined potential socio-economic, geographic and ethnicity-related mechanisms for the unintentional transport injury disparities. Each of the variables retained in the best-fitting multivariable regression model is considered in turn:

1. Proportion of the HSDA population residing in an urban area: Consistent with previous research, our findings indicate higher transport injury rates for rural populations [12–14]. Reasons for greater risk in rural areas include roads designed with less safety features (e.g., guard rails, road markings, pedestrian and cycling infrastructure) and/or in poor condition (e.g., unpaved, limited maintenance and snow removal), high speeds, long distances traveled, and distance to emergency medical services [15,37,38]. In addition, rural drivers differ in their safety attitudes and behaviours, having lower risk perceptions, engaging in riskier behavior, and perceiving safety interventions as less useful than urban drivers [39]. Past research has also confirmed lower seat belt use among Aboriginal populations, particularly while on reserves [16,40].
  A much lower proportion of the Aboriginal population in BC lives in metropolitan areas (29%) than the total population (61%), and Aboriginal people living on-reserve are less likely than those off-reserve to reside in metropolitan areas (19% versus 38%) [19]. These residential patterns are reflected in the respective SRRs reported in Table 1. The fact that this variable did not significantly interact with Aboriginal ethnicity, suggests that the increased SRR of transport injury experienced by Aboriginal populations is accounted for by the same risk factors that apply to any individual living in a rural landscape. As such, prevention strategies that target risk factors relevant to rural residents in general (e.g., engineering interventions, such as rumble strips, and median barriers; enforcement strategies, such as speed or driving while impaired checks [41]), may also be effective in reducing transport injury rates among Aboriginal populations.
2. Population per room: Our findings indicate that increased population per room was associated with decreased risk of transport injury. In BC, the average population per room is low (0.48) [13, 20, 21], thus it is possible that an increase in the population per room may be an indicator of family structure (e.g., adults with children and/or elderly relatives). One could speculate that the increased responsibility for vulnerable family members could be associated with greater care while driving. Previous research has found increased risk of transport injuries among single, divorced and widowed individuals [42].
3. Proportion of the population with a high school certificate: Higher levels of high school education were associated with decreased risk of transport injury. This finding is consistent with previous research, which also showed that individuals with lower levels of educational attainment were more likely to drive while under the influence of alcohol and less likely to use seat belts [43]. Furthermore, individuals with higher educational attainment may be able to afford newer cars with better safety standards.
4. Proportion of the population employed: While it might seem paradoxical that an indicator of improved socioeconomic prosperity would be associated with greater risk of injury, it is possible that the inclusion of other socioeconomic indicators in our analyses has already accounted for this aspect in the total population. Thus, what is represented here might be more closely related to behaviours that are associated with increased risk of transport injury, such as commuting to work and/or participating in professions that require driving (e.g., truck driver).
5. Population per room (interaction with Aboriginal ethnicity): In contrast to the finding for the total population described above, this relationship is in the opposite direction and of much greater magnitude. The average population per room among Aboriginal populations is 0.52 and 0.68 for off-reserve and on-reserve, respectively (in contrast to 0.48 for the total population) [13, 20, 21]. Because conditions for Aboriginal populations are more crowded than for the total population, an increase in this variable could be an indicator of worsening socioeconomic conditions and more pronounced disadvantage.
6. Proportion of the population employed (interaction with Aboriginal ethnicity): In contrast to our discussion above regarding the influence of employment on transport injury rates in the total population, improvement in the proportion employed in an Aboriginal community could indicate improving socioeconomic conditions that would decrease risk of injury.

To date, we have published a series of reports on Aboriginal injuries in BC stemming from our research project, *Injury in British Columbia’s Aboriginal Communities*: *Building Capacity while Developing Knowledge* [17–26][17,19–22]. This report and others that we have published on specific injury causes have illuminated important patterns in time trends that bear highlighting for the insight they provide to the living conditions of Aboriginal people in BC, as well as the etiology and prevention of these injuries. We have seen that for some categories of injury, including transport injuries and falls, injury disparities are closing. In contrast for other categories, including iatrogenic and intentional injuries, they are not. Our ecological analyses indicate that this effect is due to the nature and effect of Aboriginal ethnicity [18,19,24,26,44]. The best-fitting multivariable regression models with falls or transport injury as the outcome have Aboriginal ethnicity as a multiplicative interaction with socioeconomic factors (for falls injuries, these factors were population per room and labour force participation) [24]. Therefore, if Aboriginal ethnicity remains constant and the socioeconomic disparity diminishes, then the model predicts that the injury disparity between the Aboriginal and total populations will diminish too: which is what was observed during the period 1991–2010. In short, to close the injury gap completely in the future, it would be sufficient to close the socioeconomic gap. For example, previous research on motor vehicle crashes in Aboriginal populations has indicated the importance of various factors relevant to the socioeconomic conditions of individuals: substance use (alcohol, drugs, medications), fatigue, driving older vehicle; as well as the socioeconomic conditions of the community: road surface, level of enforcement of safety laws, availability of safety devices (e.g., car seats), provision of driver education courses, and restricted availability of alcohol [16,45–47]. More generally, our findings suggest that while efforts to improve educational attainment, housing conditions and employment rates for Aboriginal populations do not target injury prevention specifically, they would nonetheless be expected to have an important impact on reducing injury rates.

In contrast to the findings outlined above for transport or falls, with iatrogenic injury as the outcome (i.e., injuries resulting from medical treatment or therapy), Aboriginal ethnicity and socioeconomic descriptors are independent factors [26]. Therefore, if Aboriginal ethnicity remains constant and the socioeconomic disparities diminish, then these models predict that the injury disparity between the Aboriginal and total populations will diminish somewhat, but there will remain a persistent gap due to conflicting, or independent effects of Aboriginal ethnicity. This is consistent with the injury time trends. In short, to close the injury gap completely in the future, it would not be sufficient to close the socioeconomic gap. The nature and effect of Aboriginal ethnicity would also have to change. The history of Canada contains many examples of horrific attempts to modify or eliminate Aboriginal ethnicity and it should not be our aim to do so [48]. Rather, our aim should be to reduce the institutionalized racism that permeates Canadian society, such that Aboriginal ethnicity would be less of a disadvantage. The Truth and Reconciliation Commission of Canada recently released its final report with 94 recommendations to redress the colonial legacy of residential schools, many of which are directed at combatting endemic racism [49].

We have outlined the strengths and limitations associated with the data and our methods in previous publications [21,23,25]. Our population-based study is novel, not only in its population-based method and identification of Aboriginal populations, but also in using the long-form Census to obtain socioeconomic characteristics, and considering the independent effects of socioeconomic factors, geography and ethnicity on injury. While we could not link directly to the national Indian Status Registry, comparison with previous research by Vital Statistics Agency of BC suggests that our methods undercounted by about 11% [17,31]. Because the undercounting applies to both the numerator (hospitalization counts) and the denominator (population counts), this should not bias our calculated rates of hospitalization among Aboriginal people. A further limitation of our study relates to our inability to account for other relevant variables that could confound the relationships described herein. For example, we were not able to examine the influence of alcohol and other substance use, access to healthcare or trauma centers, nor whether patterns reported herein vary by injury severity.

## Conclusions

Our results indicate that significant gains have been made in reducing disparities associated with unintentional transport injuries between the Aboriginal and total populations of BC. However, Aboriginal people living on reserves and in non-metropolitan regions of the province continue to face disproportionately higher risks. There is a need to address the underlying socioeconomic disparities and endemic racism faced by Aboriginal populations, in addition to developing and implementing culturally sensitive and relevant transport injury prevention programs suited to the different geographic realities. Our findings support the BC Provincial Health Officer’s recommendations outlined in the report on motor vehicle crashes, which encourage supporting the development of community-driven research and prevention programs [34].

## Supporting information

S1 Table Standardized relative risks of hospitalization for categories of unintentional transportation injury, British Columbia, 1991–2010. (DOC)
