# Correction: Hospitalizations due to unintentional transport injuries among Aboriginal population of British Columbia, Canada: Incidence, changes over time and ecological analysis of risk markers

**DOI:** 10.1371/journal.pone.0193218

**Published:** 2018-02-15

**Authors:** Mariana Brussoni, M. Anne George, Andrew Jin, Ofer Amram, Rod McCormick, Christopher E. Lalonde

There is an error in affiliation 6 for author Ofer Amram. Affiliation 6 should be: Department of Nutrition and Exercise Physiology, Elson S. Floyd College of Medicine, Washington State University, USA.
