# Supplementary material for: Hospitalizations due to unintentional transport injuries among Aboriginal population of British Columbia, Canada: Incidence, changes over time and ecological analysis of risk markers
Source: PLoS One. 2018 Jan 26;13(1):e0191384. doi: 10.1371/journal.pone.0191384 (PMC5786298; doi:10.1371/journal.pone.0191384)
Supplement: S1 Table — (DOC) [file pone.0191384.s001.doc]

| **S1 Table. Standardized Relative Risks of hospitalization for categories of unintentional transportation injury, British Columbia, 1991-2010.** | | | | | | | |
| --- | --- | --- | --- | --- | --- | --- | --- |
|  |  |  |  |  |  |  |  |
| **Population and injury category** | **SRR^†^ 1991** | **SRR^†^ 2010** | **1991 to 2010 % change** | **p*** | **Annual % change** | **95% CI for Annual % change** | |
|  |  |  |  |  |  |  |  |
| Aboriginal population |  |  |  |  |  |  |  |
| Motor vehicle: occupant | 4.61 | 0.47 | -89.9% | 0.000 | -11.3% | -13.2% | -9.4% |
| Motor vehicle: pedal cycle rider | 5.06 | 1.31 | -74.1% | 0.819 | -6.9% | -15.8% | 3.0% |
| Motor vehicle: pedestrian | 4.78 | 0.75 | -84.3% | 0.035 | -9.3% | -14.1% | -4.2% |
| Motorcyclist | 1.13 | 0.17 | -84.6% | 0.290 | -9.4% | -14.0% | -4.5% |
| Non motor vehicle pedal cycle | 2.47 | 0.33 | -86.8% | 0.216 | -10.1% | -13.7% | -6.4% |
| Off-road motor vehicle | 1.09 | 1.50 | 37.7% | 0.335 | 1.7% | -3.1% | 6.7% |
| Other or unspecified transport. | 3.41 | 0.54 | -84.2% | 0.223 | -9.3% | -13.3% | -5.0% |
|  |  |  |  |  |  |  |  |
| BC total population |  |  |  |  |  |  |  |
| Motor vehicle: occupant | 1.80 | 0.48 | -73.2% |  | -6.7% | -7.1% | -6.3% |
| Motor vehicle: pedal cycle rider | 1.70 | 0.55 | -67.4% |  | -5.7% | -7.6% | -3.8% |
| Motor vehicle: pedestrian | 1.53 | 0.75 | -51.2% |  | -3.7% | -4.6% | -2.8% |
| Motorcyclist | 1.63 | 0.43 | -73.5% |  | -6.8% | -7.7% | -5.8% |
| Non motor vehicle pedal cycle | 1.58 | 0.34 | -78.2% |  | -7.7% | -8.5% | -6.9% |
| Off-road motor vehicle | 1.13 | 0.98 | -13.4% |  | -0.8% | -1.9% | 0.4% |
| Other or unspecified transport | 1.67 | 0.46 | -72.5% |  | -6.6% | -7.5% | -5.6% |

| **Notes:** |
| --- |
| * probability (2-sided, z-test) that Ln((SRR 2010)/(SRR 1991)) Aboriginal = Ln((SRR 2010)/(SRR 1991)) BC |
| **^†^**SRR: Standardized Relative Risk (indirectly standardized by age, gender and HSDA, compared to the total |
| population of BC, 1991 to 2010) = Observed/Expected |
|  |
|  |
